# Plasma surrogate markers of neutrophil extracellular traps correlate with disease severity in patients with moderate to severe acute respiratory distress syndrome

**DOI:** 10.1186/s12950-025-00448-8

**Published:** 2025-06-13

**Authors:** Joni A. Aoki, Frederik Denorme, Mark J. Cody, David P. Perry, John L. Rustad, Samuel M. Brown, Stephanie A. Goldstein, Elizabeth A. Middleton, Christian C. Yost, Estelle S. Harris, for the NHLBI Prevention and Early Treatment of Acute Lung Injury (PETAL) Network Investigators

**Affiliations:** 1 University of Utah School of Medicine, Salt Lake City, UT USA; 2 Department of Emergency Medicine, Washington University School of Medicine, St. Louis, MO USA; 3 Molecular Medicine Program, University of Utah, Salt Lake City, UT USA; 4 Department of Pediatrics, Division of Neonatology, University of Utah, Salt Lake City, UT USA; 5 Intermountain Medical Center, Murray, UT USA; 6 Department of Internal Medicine, University of Utah, 30 North Mario Capecchi Dr., 2nd Floor North, Salt Lake City, UT 84112 USA

**Keywords:** Acute respiratory distress syndrome (ARDS), Neutrophil extracellular traps (NETs), Surrogate markers, Cell-free DNA fluorescence assay, Plasma myeloperoxidase (MPO)-DNA complex ELISA, Clinical outcomes

## Abstract

**Background:**

Although studies have evaluated the presence of cell-free DNA and neutrophil extracellular traps (NETs) in acute respiratory distress syndrome (ARDS), the kinetics of NET formation during the early ICU admission and whether plasma NET markers correlate with clinical outcomes in patients with moderate-to-severe hypoxemia remain unknown. We sought to determine whether serial plasma NET marker levels in study participants collected over 48 h post enrollment predict disease severity and mortality in non-COVID-19 ARDS patients.

**Methods:**

We obtained previously collected plasma samples (trial enrollment, 24 h, 48 h) from 200 randomly selected ARDS participants in the completed Reevaluation of Systemic Early Neuromuscular Blockade (ROSE) Trial, as well as from 20 healthy control donors. We determined plasma levels of surrogate biomarkers for NETs using a cell-free DNA fluorescence assay and a plasma myeloperoxidase (MPO)-DNA complex ELISA. We correlated these surrogate biomarker levels with clinical outcomes from the ROSE trial study participants.

**Results:**

ROSE plasma samples demonstrated significantly higher NET levels compared to healthy donor controls. Individual study participant NET levels did not change over the forty-eight hours after trial enrollment. Higher levels of both surrogate markers correlated with fewer ventilator-free days, but only cell free-DNA correlated with mortality and higher illness severity scores.

**Conclusion:**

Surrogate markers for plasma NET levels measured in patients with moderate or severe ARDS correlate directly with adverse clinical outcomes and may serve as biomarkers for predicting severe disease. Further studies of surrogate biomarkers for NET formation in moderate-to-severe ARDS are warranted.

## Background

Direct or indirect lung injury triggers multiple inflammatory pathways leading to acute respiratory distress syndrome (ARDS). These pathways result in alveolar epithelial damage with subsequent influx of inflammatory cells and accumulation of proteinaceous fluid [1]. Direct lung injury etiologies include aspiration, inhalation, and pneumonia among others; indirect etiologies include trauma, sepsis, and blood transfusions [1–3]. Lung injury activates alveolar macrophages and releases proinflammatory signals which recruit immune cells [3]. Neutrophils respond first to such proinflammatory signals with rapid recruitment to the lungs following acute infectious or inflammatory insults. Neutrophils represent a critical component of the innate immune response and utilize multiple methods to eliminate pathogens, including phagocytosis, reactive oxygen species generation, and degranulation.

Neutrophils also form neutrophil extracellular traps (NETs) to contain infection and effect extracellular microbial killing as part of the innate immune response to infection. Through a regulated cell death response termed NETosis, a sub-set of activated neutrophils release lattices of decondensed chromatin decorated with antimicrobial factors into the extracellular space [4]. Once generated, NETs subsequently capture, kill, and prevent dissemination of microbes beyond the local site of infection, thus, decreasing the risk of a blood stream infection or sepsis. NET formation and subsequent release into the bloodstream, may also serve as scaffolding for clot propagation leading to formation of microthrombi and ischemic injury in many disease states [5].

Plasma cell-free DNA represents circulating extracellular fragments of DNA utilized in many studies as a marker of inflammation and tissue injury. Cell-free DNA is primarily composed of small fragments of nuclear and mitochondrial DNA released during many cellular processes including apoptosis, necrosis, NETosis, release of extracellular vesicles, erythroblast enucleation, and exogenous sources [6, 7]. In heathy individuals, cell-free DNA, present in small amounts, is cleared rapidly from the blood, and primarily derived from the turnover of hematopoietic cells [8]. In both health and in several disease states, including sepsis, trauma, and viral infections, the predominant component of circulating cell-free DNA is derived primarily from neutrophils and secondarily from erythroblasts [7, 9]. Cell-free DNA is elevated in many disease states, due to both increased production and insufficient clearance [7, 10].

Many studies evaluating patients with acute lung injury and ARDS have demonstrated an association of high levels of circulating plasma levels of cell-free DNA and NET markers (DNA-MPO, citrullinated histone H3 bound DNA, or DNA-neutrophil elastase) with poor outcomes including need for mechanical ventilation, severity of ARDS and mortality [11–13]. Our group recently correlated elevated plasma NET levels in hospitalized patients with COVID-19-associated ARDS with immunothrombosis, increased risk for mortality, and increased illness severity [14]. Consistent with our report on COVID-19, additional studies have shown that NET levels increase significantly between healthy controls and patients with mild and severe ARDS [14–16].

Further, the clinical course of ARDS remains highly variable, with some patients requiring non-invasive respiratory support and others requiring highly invasive support such as mechanical ventilation and extracorporeal membrane oxygenation. An ideal NET plasma marker would allow early risk stratification of a patient’s progression to ARDS and correlate with severity of illness, degree of hypoxemic respiratory failure, and overall mortality risk. Ideally, serial measurements of the same marker over time would predict which patients have the highest chance of ARDS resolution and recovery. In this study, we evaluated correlative measures of two surrogates for NET formation, cell-free DNA and MPO-DNA complex levels, as a pilot study to identify new biomarkers for ARDS severity over the first two days of illness. To the best of our knowledge, this cohort represents the largest prospective multicenter analysis of plasma NETs from intubated patients with moderate-to-severe ARDS reported to date. Here, we show that cell-free DNA and DNA-MPO levels remain elevated over the first forty-eight hours of illness and correlate with several clinical outcomes including illness severity, mortality, and ventilator-free days in patients with moderate-to-severe ARDS.

## Methods

### Aim and design

We obtained a subset of plasma samples from the Reevaluation of Systemic Early Neuromuscular Blockade (ROSE) Trial cohort [17]. The ROSE Trial, conducted through the Prevention and Early Treatment of Acute Lung Injury (PETAL) Clinical Trials Network sponsored by the National Heart, Blood and Lung Institute (NHLBI), re-examined the role of early induced paralysis in patients with moderate-to-severe ARDS defined as a PaO_2_:FiO_2_ ratio of less than 150 [17]. The study failed to demonstrate a mortality difference between the intervention and control groups resulting in early termination. However, the early patient enrollment, serial blood sampling, and collection of demographic and patient outcome data established a robust database and biorepository for use in ancillary studies, including ours. Our experiments utilized serially collected plasma samples from a subset of 200 patients randomly selected from the 1,006 patients enrolled in the ROSE Trial [17]. The trial collected plasma samples at the time of enrollment (Day 0), at twenty-four hours (Day 1) and forty-eight hours (Day 2) after study enrollment.

### Study samples

The PETAL Pathogenesis Committee approved the ancillary NET study in October of 2020, and provided the samples to the University of Utah in July of 2021. We obtained plasma samples from 200 randomly chosen study participants who survived for at least forty-eight hours [17]. The ROSE study drew the first plasma sample at the time of study enrollment (Day 0), with two additional time points at twenty-four-hour intervals (Day 1 and Day 2). The ROSE plasma samples were collected in EDTA tubes, underwent centrifugation for 10 min at 100xg, were frozen at −70 °C, and shipped on dry ice per the ROSE study sample protocol. We also collected plasma from twenty healthy donors enrolled by our laboratory using our institutional protocol at the University of Utah for blood sampling of healthy volunteers (IRB# 0051506). The PETAL Pathogenesis Committee ensured that all ROSE trial plasma samples and clinical information were de-identified prior to arrival at the University of Utah.

### High throughput cell-free DNA fluorescence assay

We quantified cell-free DNA levels as a surrogate marker for NET formation in ROSE participant plasma samples using a cell-free DNA fluorescence assay with SYTOX Green DNA dye (Invitrogen) as previously described [14, 16]. We used a pooled healthy adult plasma standard (obtained from 12 unique volunteers using IRB# 0051506) as both a positive control and standard for quantitation across assay plates. We quantified the relative fluorescence of each sample with a fluorometric plate reader using the SoftMax Pro software (Molecular Devices) and Flex Station 3 plate reader [14, 16].

### MPO-DNA ELISA assay

We detected MPO-DNA complexes as a surrogate biomarker for NET formation in plasma using an in-house MPO-DNA enzyme-linked immunosorbent assay (ELISA) [5]. We used an anti-human MPO primary antibody (Bio-Rad) as the capture antibody and a peroxidase-labeled anti-DNA primary antibody (Cell Death Detection ELISA Kit; Roche) as the detection antibody [5, 14, 15]. We used a pooled healthy adult plasma standard (obtained from 12 unique volunteers using IRB# 0051506) as both a positive control and standard for quantitation across assay plates. We reported plasma MPO-DNA complex levels as a percentage of the pooled healthy adult plasma standard, defined arbitrarily as 100%.

### Correlation with clinical data from the ROSE trial

We obtained clinical outcome data from the ROSE Trial. These data included: 90-day survival, Day 0 illness severity scores using the Sequential Organ Failure Assessment (SOFA score), Day 0 PaO_2_:FiO_2_ ratio, and the number of mechanical-ventilator-free days through 28 days.

### Statistical analysis

The data were non-normally distributed and analyzed using non-parametric testing. Mann–Whitney Statistical Rank Test was used to compare two groups of non-normally distributed data. For the correlation between cell-free DNA and MPO-DNA complex levels, we used simple linear regression. Measurements of cell-free DNA levels and MPO-DNA complex levels were log-transformed for performing statistical analyses. Logistic regression analysis was performed for the mortality analyses. Univariable linear regression analysis was applied to assess the relationship between cell-free DNA levels or MPO-DNA complex levels and clinical outcomes. We used GraphPad Prism v.10.0.3 for data analysis and statistical testing.

## Results

We examined plasma NET levels in moderate-to-severe ARDS study participants of the NIH-sponsored ROSE trial by quantifying two different surrogate markers of NET formation in plasma and correlating the results with clinical outcomes in this cohort of ARDS patients. We also quantified surrogate markers of NET formation in 20 healthy adult controls. We present the demographics for the 200 randomly chosen ROSE trial participants employed in this ancillary study as well as those of the 20 healthy adult control study participants in Table 1.

**Table 1:** Clinical characteristics of ROSE trial sample group and healthy donors

	ROSE Trial Intervention Group	ROSE Trial Control Group	ROSE Trial Sample Group	Healthy Controls
Characteristics				
Sample Size	501	505	200	20
Age, years mean ± SD	56.6 ± 14.7	55.1 ± 15.9	55.6 ± 15.6	40.1 ± 15.0
Female %	41.9	46.7	44.5	35.0
Mortality %	42.5	42.8	39.0	–-
SOFA score mean ± SD	8.7 ± 3.6	8.8 ± 3.6	8.4 ± 3.2	–-
Ventilator-free days mean ± SD	9.6 ± 10.4	9.9 ± 10.9	10.6 ± 10.7	–-
PaO2:FiO2 mmHg mean ± SD	98.7 ± 27.9	99.5 ± 27.9	93.2 ± 28.4	–-
Primary Cause of Lung Injury				
Pneumonia—no. (%)	292 (58.3)	301 (59.6)	116 (58.0)	–-
Aspiration—no. (%)	91 (18.2)	75 (14.9)	31 (15.5)	–-
Non-pulmonary sepsis—no. (%)	68 (13.6)	71 (14.1)	28 (14.0)	–-
Trauma—no. (%)	16 (3.2)	23 (4.6)	10 (5.0)	–-
Transfusion—no. (%)	13 (2.6)	7 (1.4)	4 (2.0)	–-
Other—no. (%)	21 (4.2)	28 (5.5)	11 (5.5)	–-
Direct vs. Indirect Cause of ARDS				
Direct—no. (%)	383 (76.4)	376 (74.5)	147 (73.5)	–-
Indirect—no. (%)	97 (19.4)	101 (20.0)	42 (21.0)	–-
Other—no. (%)	21 (4.2)	28 (5.5)	11 (5.5)	–-
ARDS Severity				
PaO2:FiO2 < 120 mmHg—no. (%)	362 (72.3)	358 (70.9)	157 (78.5)	–-
PaO2:FiO2 >/120 mmHg to 150 mmHg—no. (%)	139 (27.7)	147 (29.1)	43 (21.5)	–-

### Surrogate marker levels in study participants at admission and at later timepoints

**Fig. 1:**
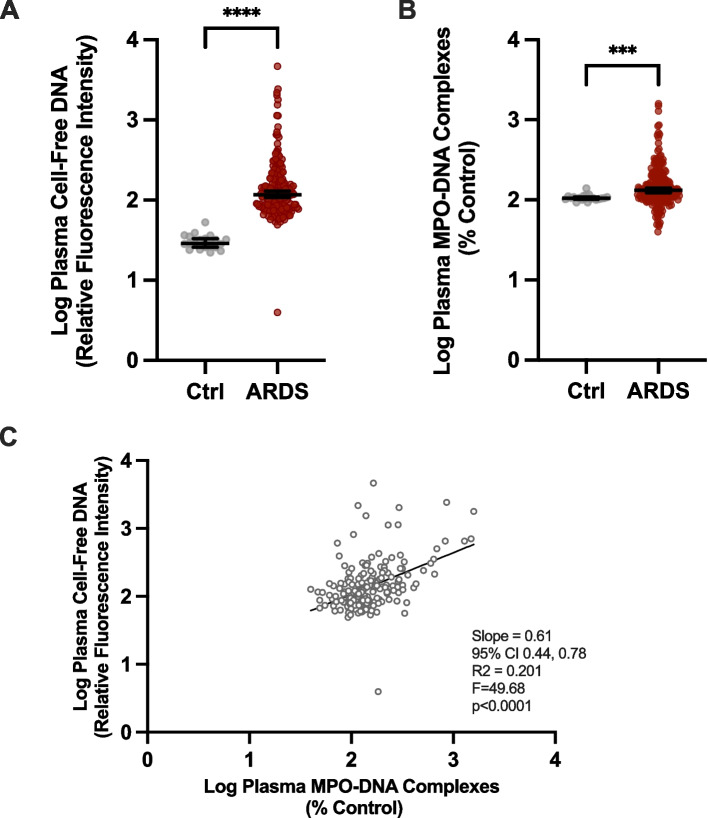
Plasma from moderate-severe ARDS participants of the ROSE trial compared with healthy controls. We determined plasma levels of surrogate biomarkers of NET formation at study enrollment in 200 ROSE trial study participants with moderate-severe ARDS and 20 healthy adult controls. **A** Cell-free DNA levels transformed logarithmically on the y-axis (median ± 95% CI) with control and ROSE trial participant groups on the x-axis. **B** MPO-DNA complex levels transformed logarithmically on the y-axis (median ± 95% CI) with control and ROSE trial participant groups on the x-axis. For (**A-B**), we employed the Mann–Whitney statistical tool. *** and **** denote *p* < 0.001 and *p* < 0.0001, respectively. **C** We performed linear regression analysis on log-transformed cell-free DNA levels (y-axis) in relation to log-transformed MPO-DNA complex levels (x-axis) in plasma samples at the time of enrollment for all 200 ROSE trial study participants included in this study

**Fig. 2:**
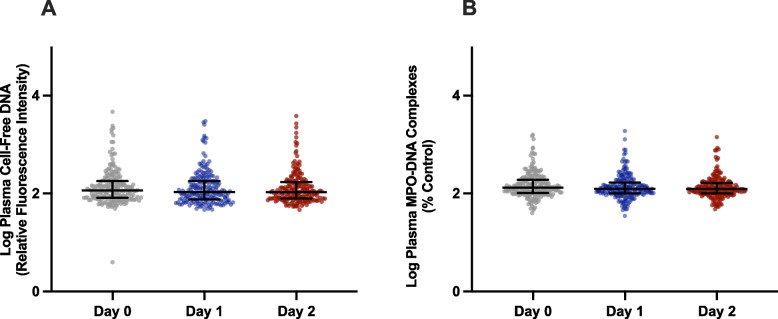
Level of plasma surrogate biomarkers of NET formation on Days 0, 1, and 2. **A** Plasma cell-free DNA levels logarithmically transformed on the y-axis (median ± 25–75% quartiles) for ROSE trial participants at study enrollment (Day 0), 24 h after enrollment (Day 1), and 48 h after enrollment (Day 2).** B** Plasma MPO-DNA complex levels logarithmically transformed on the y-axis (median ± 25–75% quartiles) for ROSE trial participants at study enrollment (Day 0), 24 h after enrollment (Day 1), and 48 h after enrollment (Day 2). We employed the Dunn’s multiple comparison test (**A-B**)

We demonstrated significantly higher median cell-free DNA and MPO-DNA complex levels at the time of enrollment (Day 0) in the ROSE trial ARDS participant samples compared to the healthy adult control participant samples (Fig. 1A-B). In these samples, median cell-free DNA and MPO-DNA complex levels correlated positively (*r* = 0.4479, *p* < 0.0001) with each other in study participants at the time of enrollment (Day 0) (Fig. 1C). Next, we determined that cell-free DNA and MPO-DNA complex levels did not change over the three time points (study days 0, 1, and 2) after study enrollment (Fig. 2A, cell-free DNA; Fig. 2B, MPO-DNA complexes). Additional analysis of median cell-free DNA and MPO-DNA complexes levels did not differ between any of the etiologies of ARDS on day 0, and this result held steady over the two additional time-points (data not shown). Similarly, median cell-free DNA and MPO-DNA complexes levels did not differ between direct or indirect causes of ARDS (data not shown).

### Associations between surrogate markers and mortality

Using logistical regression analysis of cell-free DNA levels, we detected a statically significant increase in the odds ratio (OR) for mortality with cell-free DNA levels (OR 2.58, 95% CI, 1.14–6.21) but did not see a statistically significant correlation for mortality with MPO-DNA complex levels (OR 1.9), 95% CI,0.63–5.75, (Table 2).

**Table 2:** Logistic regression of NET markers (Day 0) vs. mortality

	OR	95% CI	*p* Value
Cell-free DNA vs. mortality	2.58	1.14, 6.21	0.028
MPO-DNA complexes vs. mortality	1.90	0.64, 5.75	0.249

Table 2* Plasma surrogate markers of NET formation in relation to mortality in moderate-severe ARDS study participants*. Using logistic regression, we estimated the relationship between mean percent mortality with surrogate biomarkers of NET formation

A subsequent analysis using the absolute change (up or down) in cell-free DNA or MPO-DNA complex levels between days 0 and 2 did not show a correlation between the OR for cell free DNA levels (OD 1.0, 95% CI 0.99–1.0) or for MPO-DNA complex levels (OR 1.0. 95% CI 0.99**-** 1.0) (Table 3).

**Table 3:** Logistic regression of change in surrogate NET markers (Day 2 vs. Day 0) vs. mortality

	OR	95% CI	*p* Value
Change in cell-free DNA vs. mortality	1.00	0.99, 1.00	0.462
Change in MPO-DNA vs. mortality	1.00	0.99, 1.00	0.618

Table 3* Changes in net change in plasma surrogate markers of NET formation from Day 0 to Day 2.* The changes in plasma cell-free DNA and MPO-DNA complex levels between Day 0 and Day 2 in relation to mortality. Analysis performed using logistic regression

### Associations between surrogate markers with organ failure scores and clinical outcomes

We next assessed potential correlations of plasma surrogate biomarker levels for NET formation at baseline in ROSE trial participants with several of the trial’s prespecified clinical outcome measures [17]. Higher plasma cell-free DNA levels were associated with higher SOFA scores (Regression Coefficient 2.53, F(1,197) = 16.9, *p* < 0.0001) (Table 4) and with fewer ventilator-free days by linear regression (Regression Coefficient −8.64, F(1,120) = 11.2, *p* = 0.001) (Table 4), but detected no statistically significant differences in PaO_2_:FiO_2_ ratios with increasing cell-free DNA levels (Table 4). Likewise, higher plasma MPO-DNA complex levels showed a similar pattern and approached statistical significance for association with fewer ventilator free days (*p* = 0.06), but not higher SOFA scores or PaO_2_:FiO_2_ ratios (Table 4).

**Table 4:** Linear regression of NET markers (Day 0) vs. clinical outcome data

	Regression Coefficient	95% CI	*p* Value
Cell-free DNA vs. SOFA score	2.53	1.32, 3.74	< 0.0001
MPO-DNA vs. SOFA score	1.32	−0.39, 3.03	0.13
Cell-free DNA vs. ventilator-free days	−8.64	−13.75, −3.52	0.001
MPO-DNA vs. ventilator-free days	−5.64	−11.53, 0.26	0.06
Cell-free DNA vs. PaO2:FiO2	1.72	−9.49, 12.92	0.76
MPO-DNA vs. PaO2:FiO2	8.65	−6.56, 23.86	0.26

Table 4* Plasma surrogate markers of NET formation in relation to clinical outcomes.* We used linear regression to examine the relationship between SOFA score, ventilator-free days up to day 28, and PaO_2_:FiO_2_ ratio with surrogate biomarkers of NET formation

## Discussion

For these studies, we leveraged the robust clinical data set and plasma samples from the ROSE trial which sought to determine the efficacy of paralysis in treatment of moderate-severe ARDS defined as a PaO_2_:FiO_2_ ratio of < 150. In total, the PETAL research network recruited just over 1,000 participants meeting inclusion criteria with plasma samples collected on the day of enrollment, and additional samples collected at 24 and 48 h. Further, numerous clinically relevant outcome measures were meticulously recorded and made available to our group in a de-identified manner allowing us to accomplish the aims of our study—assessing the potential role of surrogate biomarkers of NET formation in moderate-severe ARDS. These data from 200 randomly selected ROSE trial study participants provided us by the PETAL research network, complete with serial plasma samples over 48 h and a robust, deidentified clinical outcomes database, make this, to the best of our knowledge, the largest clinical cohort interrogated for the role of cell-free DNA and MPO-DNA complex levels in ARDS patients.

We demonstrated that both cell-free DNA and MPO-DNA complex levels were elevated at the time of enrollment compared to healthy controls (Fig. 1A-B). While only the cell-free DNA levels correlated directly with mortality, baseline SOFA scores, and ventilator-free days, the results of the MPO-DNA complex assay demonstrated similar, but not statistically significant patterns (Tables 2 and 4). We expected the NET markers might not correlate with the PaO_2_:FiO_2_ ratio, due to the very low ratio of less than 150 included in the enrollment criteria for this study. Previous reports that demonstrated an excellent correlation between plasma NET levels and the PaO_2_:FiO_2_ ratios, included patients from the full spectrum of hypoxemia, with the majority being between 200 and 500 [13, 14, 18].

Cell-free DNA has long been associated with increased mortality in hospitalized critically ill patients, including in patients with sepsis and pneumonia [19, 20]. Methylation analysis studies have shown that neutrophils are the predominant cell type represented in cell-fee DNA isolated from healthy (up to 40%), exercise-stressed (> 50%), and viral respiratory infected individuals (25–50%), including influenza, COIVD-19 and human metapneumovirus [8, 21]. Cell-Free DNA isolated in our study likely includes DNA and DNA-bound fragments generated from both the inciting event (i.e., infection or trauma), secondary to acute lung injury (including non-NET related lung tissues), and additional distant organ and tissue damage, and non-host (microbe) associated DNA. Thus, it is not unexpected that in this study, the “NET-related” circulating extracellular DNA, showed less specificity for overall patient illness severity and survival. It is clear from the limited and small studies to date, that NETs levels in the lung as quantified from BAL specimens or lung tissue (obtained by biopsy or autopsy) differ from what is seen in isolated serum or plasma. NET related inflammation is likely one of many pathologic mechanisms involved in acute lung injury.

Limitations of our study include the following: small sample size, the exclusion of ARDS patients with mild illness severity and PaO_2_: FiO_2_ ratios of > 150 in the ROSE trial, and a lack of plasma sample collection beyond the 48-h time point. A small study of 18 intubated patients with moderate to severe ARDS demonstrated a significant decrease in MPO-DNA between 1–2 days compared to 5–7 days after enrollment [22], suggesting longer time-points are required to discern resolution of NET-mediated inflammation. Additional time points between 5 and 10 days would likely provide a more complete kinetic map of circulating NET markers over time. Likewise, a better understanding of the relationship between plasma and respiratory NET levels, using tracheal aspirates, bronchoalveolar lavage (BAL) fluid, or autopsy specimens would advance the understanding of a potential pathogenic role of NET formation in ARDS. We recently published a report in ARDS patients with respiratory failure secondary to COVID-19 and demonstrated increased tracheal aspirate NETs levels, compared to plasma levels when assayed from the same group of patients [14]. In addition, a recent small study comparing bronchoalveolar lavage (BAL) and serum NET levels in 18 patients with pneumonia-induced ARDS and a PaO_2_:FiO_2_ ratio of < 200 demonstrated a significant decrease in serum, but not BAL NET levels between illness days 1–2 and 5–7 [22]. Future large multicenter observational studies with comprehensive specimen collection and analysis are required to advance the field.

Based on our study and others, cell-fee DNA likely satisfies the following published properties of an ideal biomarker; they are quantifiable, can be measured by an assay that is adaptable to routine clinical practice, are reported in a timely fashion, and can be measured by a routine blood draw [23]. Our study demonstrated that plasma cell-free DNA levels were stable over at least 48 h and, thus, could be a reproducible and feasible biomarker to stratify patients at the greatest risk for poor outcomes. By contrast, the plasma MPO-DNA assay relies on an antibody capture technique that is more time-consuming, expensive, and slower to report. Future studies of ARDS will likely use cell-free DNA methylation profiles to better understand the different ARDS etiologies and subtypes [9, 21]. In summary, cell-free DNA warrants further study as a biomarker identifying ARDS patients at high risk of a poor outcome and represents a promising tool in future studies aimed at improving clinical care for ARDS patients and novel therapeutic strategies.

## Data Availability

The data for the NHLBI PETAL ROSE trial are now available through NHLBI’s repository and can be requested at https://biolincc.nhlbi.nih.gov/studies/petal_rose/. The datasets used and analyzed during the current study have not yet been placed in a public data sharing repository (they will be placed prior to publication) but are available from the corresponding author on reasonable request.
